# Tubulin Acetylation Mediates Bisphenol A Effects on the Microtubule Arrays of *Allium cepa* and *Triticum turgidum*

**DOI:** 10.3390/biom9050185

**Published:** 2019-05-11

**Authors:** Ioannis-Dimosthenis S. Adamakis, Emmanuel Panteris, Eleftherios P. Eleftheriou

**Affiliations:** 1 Department of Botany, Faculty of Biology, National and Kapodistrian University of Athens, 157 84 Athens, Greece; 2 Department of Botany, School of Biology, Aristotle University of Thessaloniki, 541 24 Thessaloniki, Greece; eelefth@bio.auth.gr

**Keywords:** bisphenol A, cytokinesis, microtubules, mitosis, tubulin acetylation

## Abstract

The effects of bisphenol A (BPA), a prevalent endocrine disruptor, on both interphase and mitotic microtubule array organization was examined by immunofluorescence microscopy in meristematic root cells of *Triticum turgidum* (durum wheat) and *Allium cepa* (onion). In interphase cells of *A. cepa*, BPA treatment resulted in substitution of cortical microtubules by annular/spiral tubulin structures, while in *T. turgidum* BPA induced cortical microtubule fragmentation. Immunolocalization of acetylated α-tubulin revealed that cortical microtubules of *T. turgidum* were highly acetylated, unlike those of *A. cepa.* In addition, elevation of tubulin acetylation by trichostatin A in *A. cepa* resulted in microtubule disruption similar to that observed in *T. turgidum*. BPA also disrupted all mitotic microtubule arrays in both species. It is also worth noting that mitotic microtubule arrays were acetylated in both plants. As assessed by BPA removal, its effects are reversible. Furthermore, taxol-stabilized microtubules were resistant to BPA, while recovery from oryzalin treatment in BPA solution resulted in the formation of ring-like tubulin conformations. Overall, these findings indicate the following: (1) BPA affects plant mitosis/cytokinesis by disrupting microtubule organization. (2) Microtubule disassembly probably results from impairment of free tubulin subunit polymerization. (3) The differences in cortical microtubule responses to BPA among the species studied are correlated to the degree of tubulin acetylation.

## 1. Introduction

Worldwide production of plastic materials has escalated over the past few decades, rendering plastic pollution a threat for human and wildlife health [1]. Neither recovery nor recycling is able to down-regulate the accumulation of plastic commodities in the environment [2]. From the macro- to nano-scale, plastics consist chiefly of different polymers, such as polyethylene, polypropylene, polyvinyl chloride, and polystyrene. Because of their large molecular size, polymers are usually considered to be biologically inert. However, because polymerization reactions are seldom complete, residual monomers or small oligomers can be found in plastic materials [3]. Their amounts may span from a few parts per million to several percentages depending on the polymer type and manufacturing process. Some of the monomers used, such as ethylene and propylene, are not considered hazardous, whereas others, such as vinyl chloride, styrene, and bisphenol A, pose risks to the biome [4].

Bisphenol A (BPA, 2,2-bis-(4-hydroxyphenyl)propane) has an annual production exceeding 3.8 million metric tons worldwide [5] and is widely used in the synthesis of various products of everyday use, including water pipes, electric and electronic equipment, thermal paper, or food containers [6,7]. As it is widely used in households and industry, BPA has been repeatedly found in wastewater effluents, raw sewage, and sewage sludge [8,9,10]. Hydrolysis of ester bonds in the plastic polymers may be the main source for BPA migration in the environment [4,11,12].

BPA, being one of the most prevalent environmental pollutants to exhibit estrogenic activity, belongs to a group known as endocrine disrupting compounds (EDCs), mimicking or blocking natural hormone action and altering the normal function of the endocrine system [13]. This “xenoestrogen” has been shown to have the capacity to alter or disrupt normal sex hormonal function in animals and humans, adversely affecting their reproductive health and fecundity [14]. Inevitably, great concern has arisen regarding the possible harmful effects from exposure of humans and wildlife to biologically active levels of BPA that may escape or be discharged into the environment [15,16].

Of particular interest are the detrimental effects of BPA on the microtubule cytoskeleton. In animal model systems, BPA promoted microtubule polymerization and centrosome-based microtubule nucleation in vitro but did not appear to display microtubule-stabilizing activities. Treatment of mammalian cells demonstrated that BPA, as well as a series of BPA derivatives, induced ectopic spindle pole formation in the absence of centrosome overduplication. Researchers postulated, therefore, that BPA hinders the nucleation of microtubules, disrupting the spatial control associated with normal chromosome segregation, resulting in aneuploidy [17,18,19,20]. Plant cell microtubule systems also appear to be targeted by BPA toxicity. In pea (*Pisum sativum*), BPA deranged interphase and mitotic microtubule arrays and promoted the formation of macrotubules, thus preventing cell division [21]. In the Greek endemic gymnosperm *Abies cephalonica*, BPA also disturbed interphase and mitotic microtubule arrays, while prometaphase, metaphase, and anaphase spindles appeared sharply pointed, sigmoid, or in multipolar conformations [22]. In maize (*Zea mays*), the effects on the microtubule arrays were similar to those observed in *Pisum sativum* plants, with taxol-stabilized microtubules appearing unaffected by BPA toxicity [23]. These studies concluded that the mitotic microtubule arrays of plants are very sensitive to BPA, as it has been reported in animal cells [24], but also that some effects of BPA are plant specific.

The objective of this study was to investigate comparatively the presumed differential effects of BPA on the microtubules and cell division of two different plant species in an attempt to elucidate the mechanism of BPA toxicity on plant microtubules. The importance of durum wheat (*Triticum turgidum*) and onion (*Allium cepa*) as cultivated crops, the profuse availability of seeds and their high germination ability, were decisive in selecting them as experimental material. Moreover, because acetylation of α-tubulin is a post-translational modification not only related to microtubule stability, but also being a key regulator of multiple cellular functions mediated by microtubules [25], we further investigated the levels of acetylated α-tubulin in these plants to reveal any correlation between α-tubulin acetylation and BPA effects. It could be hypothesized that the difference among species-specific microtubule disruption, under BPA stress, could be correlated with the acetylation status of α-tubulin.

## 2. Materials and Methods

### 2.1. Plant Material and Exposure Conditions

Caryopses of durum wheat (*Triticum turgidum* subsp. *durum* Desf. cv. Aias), kindly provided by the Cereal Institute of Thessaloniki, Greece, and seeds of onion (*Allium cepa* L. cv. Rossa Savonese), purchased from a local market, were germinated in Petri dishes on filter paper soaked with distilled water in a growth chamber at 21 ± 1 °C in the dark for 2 or 4 days, respectively. Then, the emerged seedlings were exposed to aqueous solutions of 50 mg/L BPA (Table 1), whereas other seedlings placed in distilled water were used as controls.

The concentrations of 50 mg/L BPA and exposure time were selected given that this experimental concentration was used to disrupt microtubule organization [19].

Recovery experiments were also carried out for seedlings treated with 50 mg/L BPA for 3 h, which were then transferred to distilled water and harvested for examination after 1, 3, 6, 12, 15, and 24 h.

### 2.2. Combined Treatments with BPA and Anti-Microtubule Drugs

All chemicals and reagents were purchased from Sigma (St. Louis, MO, USA), Merck (St. Louis, MO, USA), and Applichem (Darmstadt, Germany), unless otherwise stated. Seedlings of *T. turgidum* and *A. cepa* were also exposed to combinations of BPA with taxol (which stabilizes microtubules), while those of *Triticum turgidum* were additionally treated with combinations of BPA with oryzalin (which depolymerizes microtubules) [26], as shown in Table 2. The above combined treatments with taxol and oryzalin were conducted in order to examine whether microtubule dynamics interfere in microtubule responses against BPA toxicity.

### 2.3. Treatment with Trichostatin A

Trichostatin A (TSA) is an antifungal antibiotic that selectively inhibits histone de-acetylase function, thus interfering with the removal of acetyl groups from histones and non-histone proteins, such as tubulin [27,28]. We applied TSA on root tips of *A. cepa* to examine the induction of α -tubulin acetylation in combination with BPA action. Seedlings were treated with either 20 μM TSA for 3 h or with 50 mg/L BPA + 20 μM TSA for 3 h.

### 2.4. Imaging of Microtubules and Chromatin

Root tips of untreated and variously treated seedlings were excised and processed for tubulin immunostaining and DNA staining, as previously described [21,22], with modifications as follows. Root tips were fixed for 60 min in 8% (*w/v*) paraformaldehyde in PEM buffer (50 mM PIPES, 5 mM EGTA, 5 mM MgSO_4_·7H_2_O), pH 6.8, and then washed in PEM. The cell walls were digested for 40 min in 2% (*w/v*) cellulase (Onozuka R-10, Serva, Heidelberg, Germany), 2% (*w/v*) macerozyme-R10 (Serva, Heidelberg, Germany), and 0.4% (*v/v*) β-glucuronidase in PEM. Following a quick wash in PEM, the root tips were gently squashed onto polylysine-coated coverslips. The separated cells were left to dry, then extracted with 5% (*v/v*) DMSO + 5% (*v/v*) Triton-X 100 for 1 h, and incubated overnight with a rat anti-α-tubulin antibody (YOL 1/34, Serotec, Puchheim, Germany), diluted 1:80 in PEM. After washing with PEM, the cells were incubated with 1:80 FITC-anti-rat in the same buffer for 3 h at 37 °C. DNA was counterstained with 3 μg mL^−1^ propidium iodide or 10 μg mL^-1^ DAPI in PEM, and the coverslips were finally mounted in an anti-fade solution.

The specimens were examined with a Nikon D-Eclipse C1 confocal laser scanning microscope (CLSM), with an optical sectioning step of 0.20 μm or 0.30 μm. An exciter at 488 nm and a barrier at 515/30 nm and an exciter at 543 nm and a barrier at 570 nm were used for tubulin and DNA, respectively. Special care was taken in order to keep the laser beam gain equal among the different treatments. Image recording was done with EZ-C1 3.20 software according to the manufacturer’s instructions. 

Some specimens were also examined with a Zeiss Axioplan microscope, equipped with a UV source and the appropriate filters: a filter set provided with exciter solid glass filter 365 nm and barrier long-wave pass band filter 420 nm (for DAPI), and another set provided with exciter pass band filter 450–490 nm and barrier pass band filter 515–565 nm (for FITC). All the photos were taken with a Zeiss Axiocam MRc5 digital camera. Digital micrographs were processed with Adobe Photoshop with only linear settings.

### 2.5. Acetylated α-Tubulin Determination

The presence of acetylated α-tubulin was determined in untreated and variously treated roots by applying the same as above immunolocalization procedure, except that a mouse anti-acetylated α-tubulin (clone 6-11B-1) antibody [29] in dilution 1:40 was used.

Acetylated α-tubulin and total α-tubulin levels were also estimated by western blotting. Root meristem tissues of untreated and variously treated plants were homogenized at 4 °C with 1 volume of ice-cold extraction buffer (20 mM β-glycerophosphate, 20 mM HEPES, pH 7.5, 20 mM NaF, 2 mM EDTA, 0.2 mM Na_3_VO_4_, 10 mM benzamidine, 5 mM DTT, 1% (*v/v*) Triton X-100, and a commercial protease inhibitor cocktail). Lysates were incubated on ice for 10 min and then centrifuged at 4 °C, for 10 min at 16,000 *g*. The supernatants were mixed proportionally with Laemmli sample buffer and subjected to SDS–PAGE. Approximately, 100 μg of protein was loaded per well. The protein content was determined by Bradford assay. Gels were semi-dry blotted to nitrocellulose membranes, which were then blocked in 5% (*w/v*) non-fat milk powder in TBST buffer (1xTBS, 0.1% (*v/v*) Tween 20) overnight at 4 °C. After washing with TBST (3 × 5 min), the membranes were incubated for 2 h at room temperature with primary antibodies diluted in TBST buffer containing 5% (*w/v*) non-fat milk powder. Primary antibodies were diluted as follows: anti-acetylated α-tubulin 6-11B-1, anti α-tubulin (DM1A, Santa Cruz Biotechnology, Santa Cruz, CA, USA), both at 1:2000, and anti-GAPDH at 1:10,000 as a loading control. Following 2 × 5 min washes in TBSTXS (1x TBS, 1% BSA, 1% Triton X, and 1% SDS), one wash with a 0.8% (*w/v*) NaCl solution, and finally, an incubation of 5 min with a 5% (*w/v*) of non-fat milk solution in TBST, the membranes were incubated for 2 h at room temperature with horseradish peroxidase-conjugated secondary antibodies (Santa Cruz) (1:2500) diluted in TBST buffer containing 5% (*w/v*) non-fat milk powder. After washing in TBSTXS buffer (3 × 5 min) and one wash with a 0.8% (*w/v*) NaCl solution, proteins were detected using enhanced chemiluminescence (Cell Signalling, Leiden, The Netherlands).

## 3. Results

### 3.1. BPA Effects on Microtubules in Interphase and Mitotic Root Cells

**Figure 1:**
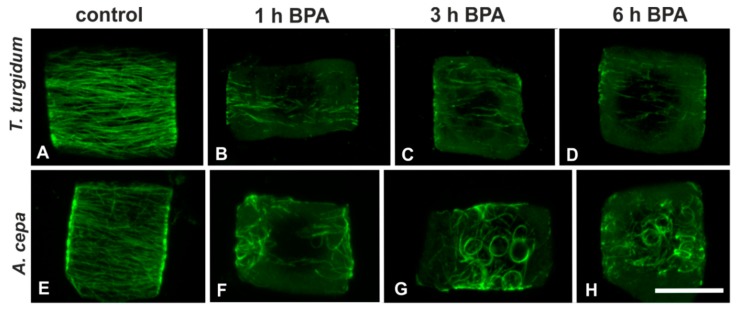
Tubulin immunolocalization in interphase root cells of the two plant species studied, either untreated or bisphenol A (BPA)-treated (as depicted), at a single cortical confocal laser scanning microscope (CLSM) section (**A**, **B**, **E**, **G**) or a maximum projection of serial sections (**C**, **D**, **F**, **H**). The plant species and treatment regime are similarly noted in all the following figures. Scale bar: 10 μm.

Interphase cells of untreated *T. turgidum* and *A. cepa* roots displayed densely arranged transverse cortical microtubules (Figure 1A,E). BPA effects on interphase microtubules were readily manifested in both species upon 1 h of treatment. In *T. turgidum* roots treated with 50 mg/L BPA for 1 h, cortical microtubules of interphase cells seemed to be depolymerized (Figure 1B). The effect was similar at 3 h and 6 h treatments (Figure 1C,D). In *A. cepa* roots, treated with 50 mg/L BPA for 1 h, cortical microtubules appeared distorted and partially bundled (Figure 1F), while at 3 h and 6 h treatments, curly, wavy, and ring-like tubulin structures were encountered (Figure 1G,H). 

The effect of BPA on mitotic cells was studied step by step, following the normal sequence of the cell cycle. Perturbations on microtubules and chromatin/chromosome morphology were co-investigated, since they supplement each other in recognizing the cell cycle stages. However, in BPA-treated cells it was frequently difficult to determine the exact stage of each cell, due to severe disturbance of the mitotic events and uncoupling of microtubule organization from chromosome morphology. 

**Figure 2:**
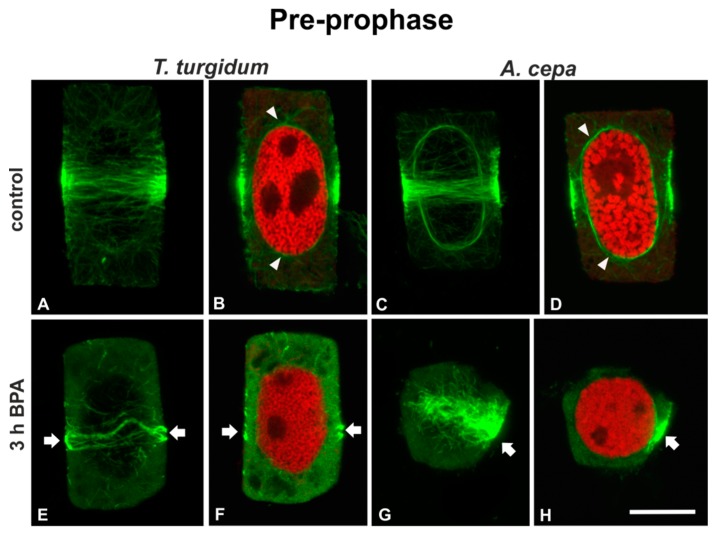
(**A**,**C**,**E**,**G**) Tubulin immunolocalization (green, projections of CLSM sections) and (**B**,**D**,**F**,**H**) propidium iodide DNA staining (red, single CLSM sections) in pre-prophase root cells. Arrows point to aberrantly organized pre-prophase microtubule bands of BPA-affected cells. Note the absence of perinuclear microtubules in (**F**) and (**H**) in contrast with the control (arrowheads in (**B**,**D**)). Scale bar: 10 μm.

Pre-prophase cells of untreated roots of both species displayed a typical broad pre-prophase microtubule band and microtubules surrounding the nucleus periphery (Figure 2A–D). Pre-prophase cells of roots treated for 1 h (data not shown) or 3 h with 50 mg/L BPA exhibited pre-prophase bands with atypical microtubule arrangement, while perinuclear microtubules were absent (Figure 2E–H). In particular, *T. turgidum* pre-prophase cells exhibited diminished and distorted pre-prophase bands and very scarce perinuclear microtubules (Figure 2E,F), while similarly treated pre-prophase root cells of *A. cepa* bore unilaterally compact microtubule bands and faint dispersed microtubules on the other side, with no perinuclear microtubules at all (Figure 2G,H).

**Figure 3:**
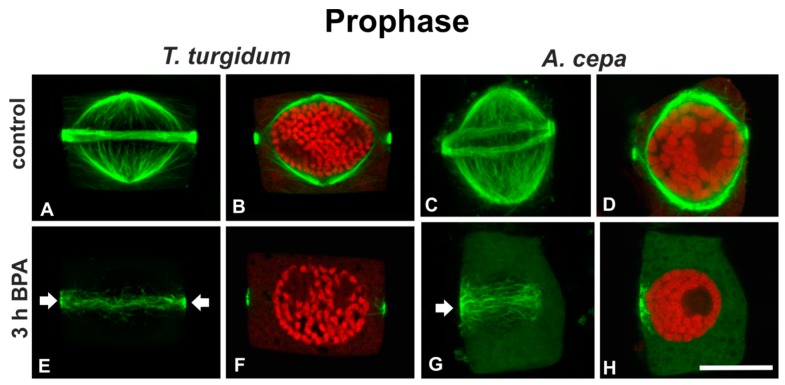
(**A**,**C**,**E**,**G**) Tubulin immunolocalization (green, projections of CLSM sections) and (**B**,**D**,**F**,**H**) propidium iodide DNA staining (red, single CLSM sections) in prophase (see chromatin condensation) root cells. Arrows in (**E**,**G**) point to aberrantly organized pre-prophase microtubule bands of BPA-affected cells. Note the absence of perinuclear spindles in (**F**,**H**). Scale bar: 10 μm.

Prophase cells of untreated roots had typically organized narrow pre-prophase bands and perinuclear prophase spindles (Figure 3A–D), while prophase cells of BPA-treated *T. turgidum* roots displayed degrading pre-prophase bands (Figure 3E) and those of *A. cepa* exhibited disrupted unilateral pre-prophase bands (Figure 3G). In prophase cells of both plants, prophase spindles were absent (Figure 3F,H). The above effects were obvious even at short treatments (1 h; data not shown).

**Figure 4:**
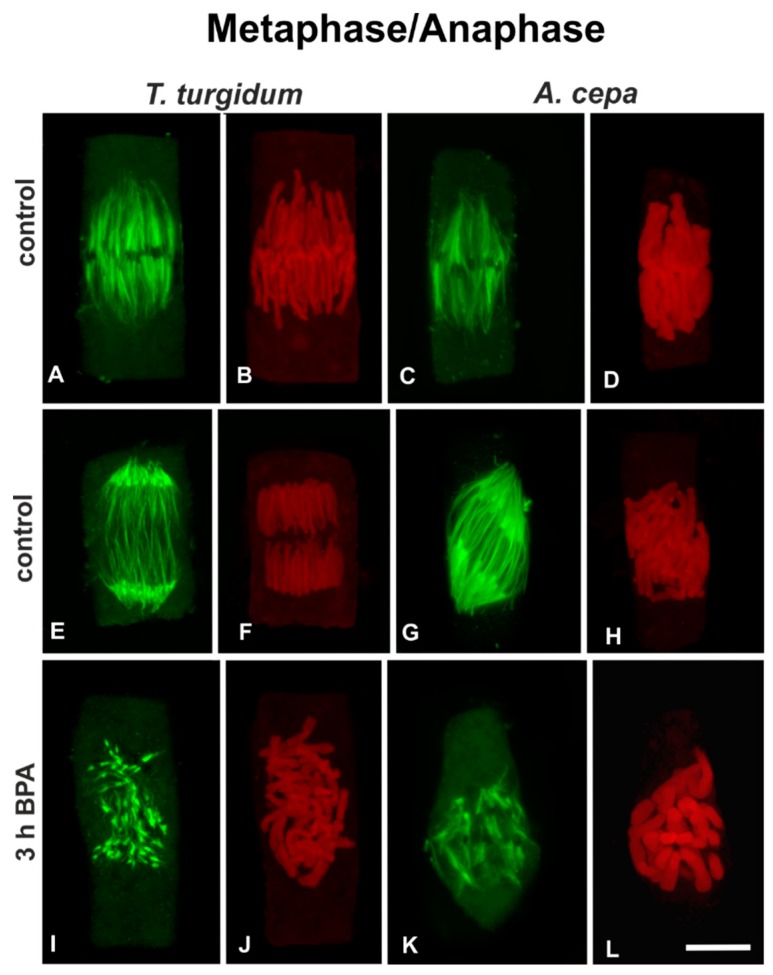
(**A**,**C**,**E**,**G**,**I**,**K**) Tubulin immunolocalization (green) and (**B**,**D**,**F**,**H**,**J**,**L**) propidium iodide DNA staining (red) in untreated metaphase (**A**–**D**) and anaphase (**I**–**L**) cells, as well as BPA-affected mitotic **I**–**L**) root cells. In BPA-treated cells the metaphase or anaphase status could not be distinguished. All images are projections of CLSM sections. Scale bar: 10 μm.

Metaphase cells of untreated roots of both species exhibited typical spindles, the kinetochore bundles of which converged to either sides of the equatorial plane (Figure 4A,C), where the kinetochores were arranged (Figure 4B,D). Cells of untreated roots of both species at anaphase (Figure 4E–H) displayed typical anaphase spindles, consisting of shortened kinetochore microtubules and elongated interzonal microtubules (Figure 4E,G), while sister chromatid groups were separating (Figure 4F,H). After 3h of treatment with 50 mg/L BPA, in both species, typical metaphase or anaphase cells could not be distinguished. On the contrary, mitotic cells with severely disrupted spindles, consisting of short dispersed kinetochore microtubule bundles (Figure 4I,K) among disorderly aggregations of chromosomes (Figure 4J,L) were found. Similar effects were observed after treatments for 1 h or 6 h (data not shown).

**Figure 5:**
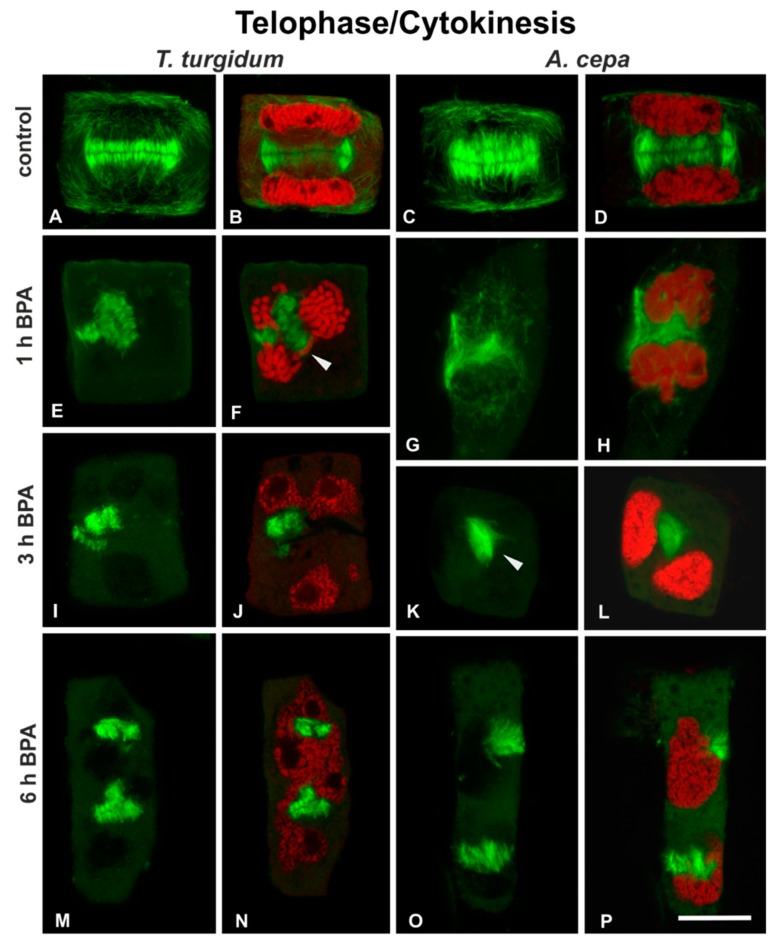
(**A**–**P**) Tubulin immunolocalization (green) and (**B**,**D**,**F**,**H**,**J**,**L**,**N**,**P**) propidium iodide DNA staining (red) in telophase/cytokinetic root cells. Arrowhead in (**F**) points to a lagging chromosome arm, while arrowhead in (**K**) points to unexpanded phragmoplast. Note the extensive presence of branched phragmoplast conformations (**E**–**J**,**M**,**N**). (**A**,**C**,**E**,**G**,**H**,**I**,**K**,**M**,**O**) Maximum projections of CLSM sections; (**B**,**D**,**F**,**J**,**L**,**N**,**P**) single sections. Scale bar: 10 μm.

Telophase/cytokinetic cells of untreated roots displayed typical phragmoplasts (Figure 5A–D) developing between the daughter nuclei (Figure 5B,D). In cells of both species treated with 50 mg/L BPA for 1 h and 3 h, the phragmoplasts appeared strongly affected, disordered, branched, misaligned, or compacted (Figure 5E–L), located between abnormally segregated and atypically positioned daughter nuclei (Figure 5F,H,J,L). After 5 h of treatment, phragmoplasts were discontinuous and fragmented (Figure 5M–P), and the daughter nuclei were atypically separated in both species (Figure 5N,P). These distorted phragmoplasts, as revealed by immunofluorescence, were a first and strong indication of defective cytokinesis.

To sum up, in both plant species, BPA treatment was fatal for the cell cycle: depending on the exact stage, in which each cell was entering while affected by BPA, the chromosomes failed to aggregate at the metaphase plate, to segregate and move to opposing poles at anaphase (Figure 4I-L) and to form the new nuclei at telophase, while cytokinesis was dramatically disrupted (Figure 5E-P). Since typical bipolar spindles were not organized under BPA treatment, metaphase could not be distinguished from anaphase in most mitotic cells.

### 3.2. Combined Treatments with Anti-Microtubule Drugs and BPA

**Figure 6:**
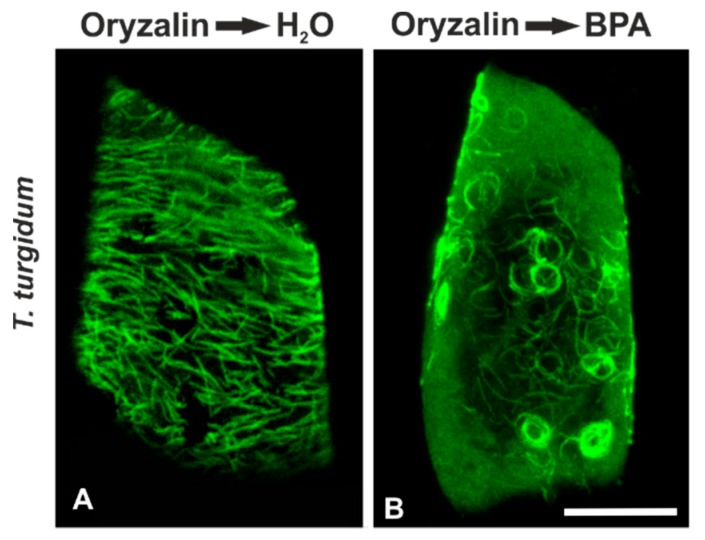
Maximum projections of CLSM sections, after tubulin immunolocalization in interphase; *T. turgidum* root cells recovering after oryzalin treatment, either in (**A**) water or in (**B**) BPA solution. Scale bar: 10 μm.

In order to decipher the possible role of microtubule dynamics in their response to BPA, we performed combined treatments with oryzalin (microtubule depolymerizing) or taxol (microtubule stabilizer) and BPA. *T. turgidum* root cells firstly treated with 5 μΜ oryzalin for 12 h and then left to recover in water for 12 h bore densely arranged but largely misaligned cortical microtubules (Figure 6A). When seedlings were first exposed to 5 μM oryzalin and then to 50 mg/L BPA for 12 h, they exhibited bundles of curly, wavy, and ring-like cortical tubulin structures (Figure 6B), resembling those encountered in BPA-affected interphase cells of *A. cepa* (*cf.* Figure 1G,H).

**Figure 7:**
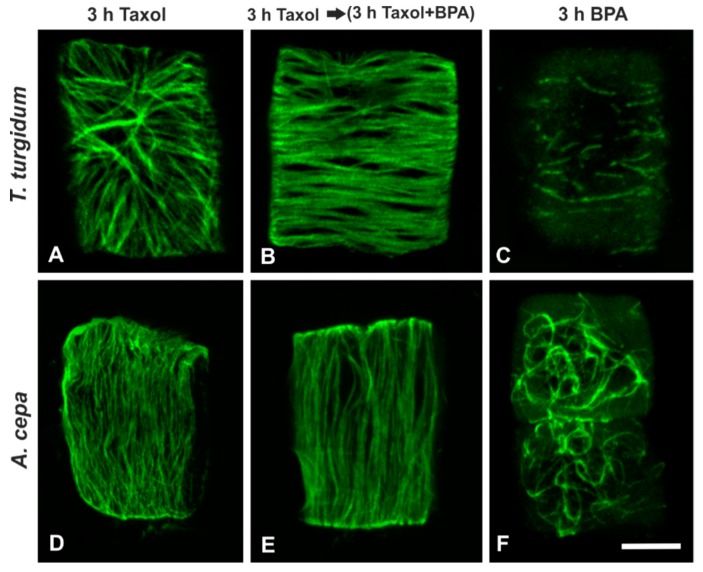
Maximum projections of CLSM sections, after tubulin immunolocalization in interphase root cells treated with taxol and/or BPA (as noted on images **A**–**F**). Scale bar: 10 μm.

Cortical microtubules of meristematic root cells in both species, subjected either to treatment with 20 μΜ taxol for 3 h (Figure 7A,D) or to 20 μΜ taxol for 3 h and afterwards to 50 mg/L BPA + 20 μΜ taxol for 3 h (Figure 7B,E) displayed an intensified array of cortical microtubules that did not resemble that observed in cells treated with BPA alone (*cf.* Figure 7C,F).

### 3.3. Presence of Acetylated α-Tubulin in the Microtubules of Interphase and Dividing Cells

**Figure 8:**
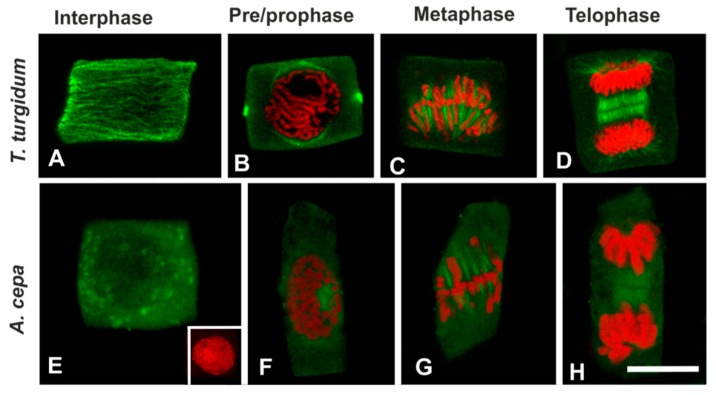
(**A**–**H**) Acetylated α-tubulin immunolocalization (green) and (**B**–**H**) propidium iodide DNA staining (red) in untreated root cells. The inset in (**E**) depicts the interphase nucleus of the cell. All images are single CLSM sections. Scale bar: 10 μm.

**Figure 9:**
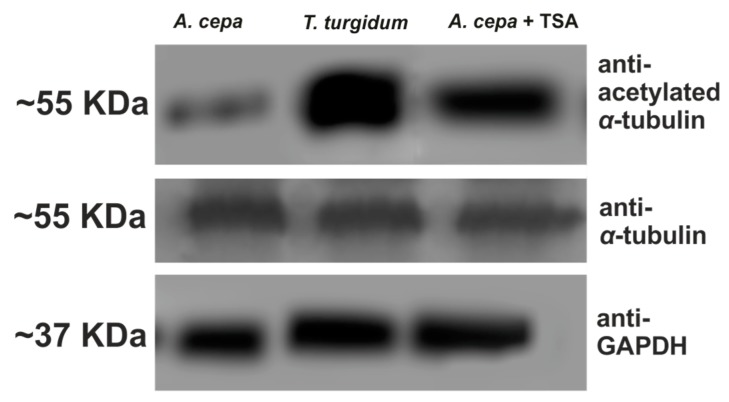
Western blot depicting the content of acetylated and total α-tubulin in roots. GAPDH is used as a loading control. TSA: trichostatin A.

In a further attempt to interpret the difference in microtubule response between the two species, the presence of acetylated α-tubulin, an indicator of stable microtubules [30], was investigated. In untreated roots of *T. turgidum*, cortical microtubules of interphase cells (Figure 8A), as well as the pre-prophase microtubule band, perinuclear microtubules, mitotic spindle, and phragmoplast consisted of acetylated α-tubulin (Figure 8B–D). On the contrary, in untreated *A. cepa* root cells no acetylated α-tubulin could be observed in interphase and pre-prophase/prophase cells (Figure 8E,F), while acetylated α-tubulin was faintly present only in mitotic and telophase/cytokinetic cells (Figure 8G,H) cells. The difference in acetylated α-tubulin content, among the two plant species, was also confirmed by western blotting (Figure 9). Total α-tubulin in both species was also examined by western blotting and remained equal in both species, also under TSA treatment (Figure 9).

### 3.4. Combined Treatments with TSA and BPA

**Figure 10:**
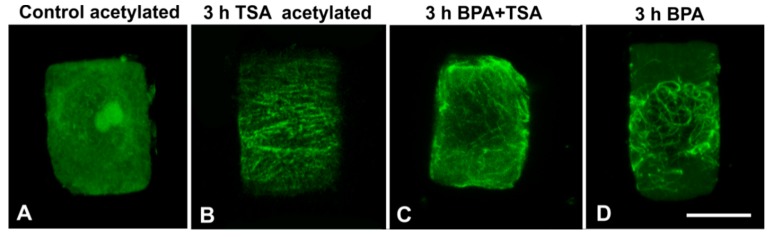
Maximum projections of CLSM sections, after acetylated α-tubulin (**A,B**) or total α-tubulin (**C,D**) immunolocalization in control and variously-treated (as noted) *A. cepa* interphase root cells. Scale bar: 10 μm.

In *A. cepa*, treatment with 20 μM TSA for 3 h resulted in a prominent increase in acetylated α-tubulin content (Figure 9). This increase was also verified by immunofluorescence microscopy in interphase cells (Figure 10A,B). Upon combined treatment with TSA + BPA for 3 h, *A. cepa* interphase root tip cells displayed a degraded microtubule network (Figure 10C), unlike the tubulin network observed after treatment with BPA alone (Figure 10D) but similar to that of BPA-treated *T. turgidum* root tip cells (*cf.* Figure 1B–D).

### 3.5. Recovery Experiments

**Figure 11:**
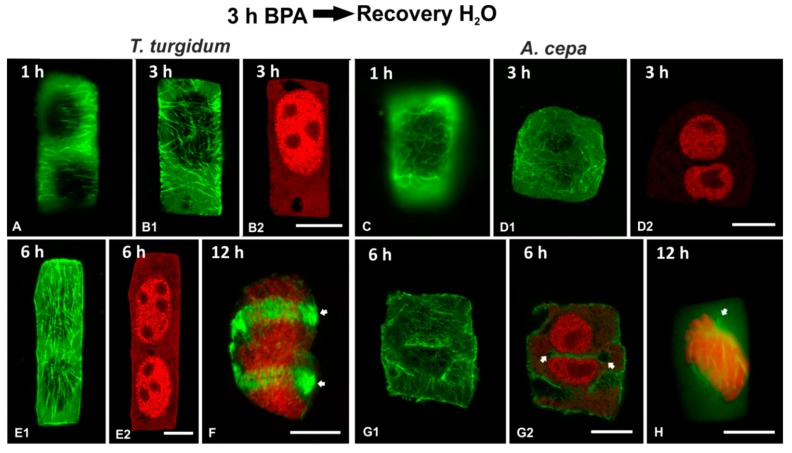
Tubulin immunolocalization (green, **A**, **B1**, **C**, **D1**, **E1**, **F**, **G1**, **H**) and propidium iodide DNA staining (red, **B2**, **D2**, **E2**, **F**, **G2**, **H**) in root cells recovering after BPA treatment for duration noted on the figures. Arrows in (**F**) point to an atypical phragmoplast, while arrows in (**G2**) point to cell plate remnant in a post-cytokinetic cell. Images (**D1**, **F**, **H**) are maximum projections of CLSM sections, while the rest of the images are single CLSM sections. Scale bars: 10 μm.

**Figure 12:**
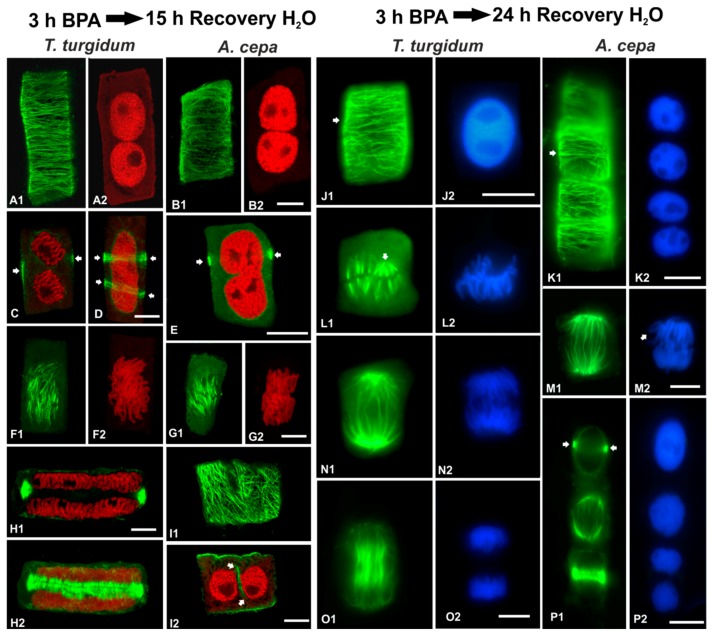
Tubulin immunolocalization (green, **A1**, **B1**, **C–E**, **F1**, **G1**, **H**, **I**, **J1**, **K1**, **L1**, **M1**, **N1**, **O1**, **P1**), propidium iodide DNA staining (red, **A2**, **B2**, **C–E**, **F2**, **G2**, **H**, **I2**) and DAPI DNA staining (blue, **J2**, **K2**, **L2**, **M2**, **N2**, **O2**, **P2**) in root cells recovering after BPA treatment. Arrows in (**C–E**) point to pre-prophase band profiles, arrows in (**I2**) point to cell plate remnant in post-cytokinetic cell, arrows in (**J1**) and (**K1**) point to interphase microtubules, arrow in (**L1**) points to a metaphase mini-pole, arrow in (**M2**) points to chromosome arms diverging laterally, while arrows in (**P1**) point to pre-prophase band profiles. Images (**A**, **B**, **F**, **H2**) depict maximum projections of CLSM sections, images (**C–E**, **G**, **H1**, **I**) are single CLSM sections, while images **(J–P**) are conventional fluorescence micrographs. Scale bars: 10 μm.

In order to examine the persistence of BPA effects on root cells, recovery experiments were performed in roots treated for 3 h in 50 mg/L BPA and then transferred to distilled water for 1, 3, 6, 12, 15 and 24 h. In both species studied, the microtubule network was able to recover when BPA was removed from the medium (Figure 11 and Figure 12). However, at least 15 h were required until microtubule organization appeared similar to those of untreated root cells (Figure 12A–I). Only after 24 h of recovery, microtubule organization was identical to that of untreated cells (Figure 12J–P).

In roots of both species, recovery periods under 15 h (1, 3, 6, and 12 h) were not enough to alleviate the deleterious effects of BPA (Figure 11). In both interphase (Figure 11 A–E,G) and dividing (Figure 11F,H) cells, microtubule organization differed significantly from that of untreated cells. In addition, binucleate cells (Figure 11D,E) and cells with remnants of incomplete cell plates (Figure 11G) were frequent.

In both species, roots that recovered for 15 h bore binucleate cells at interphase (Figure 12A–B,I) and pre-prophase/prophase (Figure 12C,D) or cells with not fully divided nuclei (Figure 12E). Pre-prophase cells displayed one (Figure 12C,E) or even two pre-prophase bands (Figure 12D). In mitotic cells, the spindles achieved an almost typical organization (Figure 12F,G), while typical phragmoplasts were present in cytokinetic cells (Figure 12H). Post-cytokinetic cells often exhibited typical cortical microtubules (Figure 12I1), while “floating” cell plate remnants lined by microtubules (Figure 12I2) were encountered between the daughter nuclei, most probably a result of aberrant cytokinesis during the BPA treatment that preceded recovery. In roots of both species that recovered for 24 h, typical interphase (Figure 12J,K) and mitotic (Figure 12L–P) cells were encountered. Only slight aberrations in either metaphase spindle (Figure 12L1) or chromosome organization were found (Figure 12L2,M2).

## 4. Discussion

A primary response of plant cells to various external stimuli, among which include several abiotic stress factors, is the alteration of microtubule conformation [31,32,33,34,35]. The results derived from both *A. cepa* and *T. turgidum* confirm that, during both interphase and cell division, microtubule structure and organization are sensitive to BPA, in agreement with previous studies [21,22,36]. Thus, it seems to be well established that, regardless of the plant species or experimental setup, a general feature of plant microtubules is the rapid and sensitive response to BPA toxicity. Nevertheless, microtubules were able to recover upon BPA removal, but required 24 h to acquire the control conformations (Figure 11 and Figure 12). However, the binucleate cells formed during BPA exposure remained and proceeded to mitosis that could lead to aneuploidy, a feature common in the presence of BPA [18].

Interestingly, microtubule array response was significantly different among the two species studied. In particular, under BPA treatment, cortical microtubules and the pre/prophase band of *T. turgidum* appeared to disintegrate (Figure 1B–D), while in *A. cepa* they were substituted by wavy, curly, and ring-like tubulin structures (Figure 1F–G). In parallel, the content of acetylated *α*-tubulin differed significantly among the two plant species. *α*-Tubulin acetylation at K40 is a common feature in angiosperms [30], so this observation is in accordance with previous studies on both *A. cepa* [37] and *T. turgidum* [38]. However, acetylated *α*-tubulin was not present in all the microtubule arrays of *A. cepa,* being absent from interphase and pre/prophase cells (Figure 8). It could be supposed that differential tubulin acetylation between the two species correlates to each specific effect of BPA on microtubule integrity and/or organization. TSA experiments further strengthen the above concept. TSA selectively inhibits histone deacetylase (HDAC) function [27,28]. HDAC was found to interact with the microtubule plus-end tracking protein EB1, suggesting that HDAC might be funneled to the lumen at this entry site [39]. A more recent report further showed that incubation of acetylated microtubules with recombinant HDAC results in random deacetylation all along the length of microtubules [40]. Therefore, HDAC inhibition increases tubulin acetylation in microtubules via inhibition of their deacetylation. When *A. cepa* microtubules were over-acetylated by TSA, they responded similar to those of *T. turgidum* and appeared depolymerized (Figure 10).

Another finding, further confirming a role for tubulin acetylation in the differential responses among the two species studied, was that the microtubule arrays of *A. cepa* that were acetylated (metaphase/anaphase spindle and phragmoplast) responded similarly to the corresponding microtubule arrays of *T. turgidum*, not forming bundles or ring-like structures. In general, post-translational modifications of tubulin, such as acetylation, de-tyrosination, and phosphorylation, play important roles in regulating the stability and function of microtubules [41]. α-Tubulin acetylation is among the most common modifications [42] and is considered to be a marker of stable microtubules and resistant to turnover [43]; although, tubulin acetylation has been also found on dynamic microtubules [25]. It is suggested that tubulin acetylation weakens lateral interactions between protofilaments, thus “softening” microtubules and increasing their flexibility [44]. That would allow microtubules to better resist mechanical stress, consequently persisting for longer time [45]. The increased content of acetylated α-tubulin in *T. turgidum* could therefore indicate that microtubule arrays of this species are more long-lived than the microtubule arrays of *A. cepa*. 

The question that then arises is how this difference in microtubule stability could explain the difference in their responses to BPA. It has been proposed that acetylation, similar to tyrosination/detyrosination modifications, may influence the transport and binding of microtubule-associated proteins (MAPs) to selected microtubules [46,47]. Therefore, a difference in MAP proteins bound to the microtubule arrays, due to the different degree of α-tubulin acetylation, could justify the differential response observed among the two plant species studied. This assumption, however, deserves further investigation. More recently, it has been found that BPA resulted in a mis-localization of MAPs, leading to a failure of spindle attachment on the kinetochores [20]. In our experiments, the contact of microtubules to kinetochores also seemed to be disrupted (Figure 4), so a reasonable assumption could be that BPA perturbed MAP localization also in plant cells, which is vital for spindle microtubule attachment to kinetochores [48]—a hypothesis deserving further confirmation.

As yet, the exact mechanism of noxious BPA action toward plant microtubules is not well understood. Among other alternatives, a mechanism based on a direct interaction of BPA with tubulin subunits has been proposed [19,21]. An interesting finding of this study, in support of the above notion, was that overstabilization of microtubules by taxol “protected” microtubule arrays of both species against BPA effects (Figure 7). This feature was prominent in other species as well [23]. Microtubule dynamics seem, thus, to be crucial for their response to BPA. Taxol lowers the critical dimer concentration for polymerization, promoting microtubule assembly in the absence of MAPs and GTP interacting with tubulin dimers [49]. Because BPA cannot interact with stabilized microtubules, it may be assumed that its effect may require binding to free dimers. Another finding further consolidating the concept of a direct BPA-tubulin dimer interaction is that in *T. turgidum*, when oryzalin-depolymerized microtubules were left to recover in the presence of BPA, wavy and ring-like conformations were organized (Figure 6). The above result is in accordance with in vitro experiments in human fibroblasts, where a direct effect of BPA on tubulin was obtained by observing that BPA interacts irreversibly with tubulin, resulting in the appearance of ring-like tubulin conformations [50].

## 5. Conclusions

In conclusion, BPA is a disruptor of mitosis/cytokinesis in a wide variety of plants. Experimental evidence supports that the detrimental effect of BPA on microtubules may occur by direct binding to free tubulin subunits, impairing the assembly of normal and functional polymers. The differences in cortical and pre/prophase microtubule array responses to BPA between the species studied here are correlated to the degree of α-tubulin acetylation. It appears, therefore, that although acetylated microtubules may be targeted by BPA, its specific effect is diversified due to the degree of tubulin acetylation.

## Figures and Tables

**Table 1:** Exposure of seedlings to bisphenol A (BPA) in hours.

BPA	*Triticum turgidum*	*Allium cepa*
50 mg/L	1, 2, 3, 6	1, 2, 3, 6

**Table 2:** Combined treatments with BPA and anti-microtubule drugs.

Plant	Treatment	Post-Treatment
*Allium cepa*	50 mg/L BPA + 20 μM taxol, 3 h	----
*Triticum turgidum*	50 mg/L BPA, 3 h 20 μM taxol, 3 h 50 mg/L BPA + 20 μM taxol, 3 h 5 μM oryzalin, 12 h 5 μM oryzalin, 12 h	50 mg/L BPA + 20 μM taxol, 3 h 50 mg/L BPA + 20 μM taxol, 3 h ---- 50 mg/L BPA, 12 h H_2_O, 12 h
